# Before and after COVID-19: Changes in symptoms and diagnoses in 13,033 adults

**DOI:** 10.1371/journal.pone.0286371

**Published:** 2024-03-08

**Authors:** Mark J. Butler, Codruta Chiuzan, Heejoon Ahn, Michael Gao, Stefani D’Angelo, Jackson Yeh, Karina Davidson

**Affiliations:** 1 Institute of Health System Science, Feinstein Institutes for Medical Research, Northwell Health, New York, NY, United States of America; 2 Donald and Barbara Zucker School of Medicine at Hofstra/Northwell, Northwell Health, Hempstead, NY, United States of America; Waseda University: Waseda Daigaku, JAPAN

## Abstract

**Background:**

Most patients with COVID-19 report experiencing one or more symptoms after acute infection subsides, known as post-acute sequelae of SARS-CoV-2 infection (PASC). Though research has examined PASC after acute COVID-19, few studies have examined PASC over a longer follow-up duration or accounted for rates of symptoms and diagnoses before COVID-19 infection, and included those not actively seeking treatment for PASC. To determine what symptoms and diagnoses are occurring at higher rates after acute COVID-19 infection from a more inclusive sample, we extracted electronic hospital records (EHR) data from 13,033 adults with previously known diagnoses and symptoms.

**Methods:**

The sample was comprised of patients who had a positive PCR test for SARS-CoV-2 between March 1, 2020, and December 31, 2020, and follow-up was conducted through November 29, 2021. All patients in the sample had medical appointments ≥4 weeks before and ≥4 weeks after their positive PCR test. At these appointments, all ICD-10 codes recorded in the EHR were classified into 21 categories based on the literature and expert review. Conditional logistic regression models were used to quantify the odds of these symptoms and diagnostic categories following COVID-19 infection relative to visits occurring before infection. The sample was comprised of 28.0% adults over 65 and was 57.0% female. After the positive PCR test, the most recorded diagnoses and symptoms were dyspnea and respiratory failure, myositis, musculoskeletal pain/stiffness, anxiety, and depression.

**Results:**

Results from regression analyses showed increased odds of diagnosis for 15 of the 21 categories following positive PCR. Relative to pre-COVID, the diagnoses and symptoms with the greatest odds after a positive PCR test were loss of smell or taste [OR (95% CI) = 6.20 (3.18–12.09)], pulmonary fibrosis [3.50 (1.59–7.68)], and dyspnea/respiratory failure [2.14 (1.92–2.40)]. Stratification of these analyses by age, gender, race, and ethnicity showed similar results.

**Conclusion:**

The increased symptoms and diagnoses detected in the current study match prior analyses of PASC diagnosis and treatment-seeking patients. The current research expands upon the literature by showing that these symptoms are more frequently detected following acute COVID-19 than before COVID-19. Further, our analyses provide a broad snapshot of the population as we were able to describe PASC among all patients who tested positive for COVID-19.

## Introduction

Due to the complex and diverse effects of coronavirus disease 2019 (COVID-19) on multiple organs and body systems [1], up to 75% of patients infected with severe acute respiratory syndrome coronavirus 2 (SARS-CoV-2) report experiencing one or more symptoms after acute infection subsides. [1] These symptoms, described both as the post-acute sequelae of SARS-CoV-2 infection (PASC) [2] and “long covid [3],” can be debilitating for patients and cause impaired health long after the acute effects of COVID-19 have subsided [1–7]. Research has examined what symptoms and diagnoses present most often among patients with PASC after acute infection. The symptoms of PASC encompass multiple classes of diagnoses including respiratory (e.g. cough, shortness of breath), circulatory (e.g. palpitations), gastrointestinal (e.g. GERD), and pain (e.g. myalgia) [1,2,4,8]. Other long-term symptoms can include prolonged loss of smell, fatigue, malaise, sleep disorders, and mental health issues [1,2,8]. These findings suggest as acute COVID-19 has a diverse symptom presentation, the long-term consequences of SARS-CoV-2 infection are equally diverse.

Still, there is less information about symptoms and diagnoses over longer follow-ups [2,9]. Many analyses of PASC examine longer term consequences over shorter periods, such as 30 days [2,9] up to 120 days [8]. This lack of information prevents identification of symptoms which may appear up to a year after initial infection. Even studies with longer follow-up durations have been unable to account for patient symptoms and diagnoses before initial SARS-CoV-2 infection [6], though some recent research has accounted for differences in patients at the time of PCR testing [8]. Knowing the prior rates of symptoms and diagnoses before a PCR test can aid in describing excess rates of diagnoses following a COVID-19 infection. By examining patients before and after infection with SARS-CoV-2, we hope to utilize the medical history of patients in a broad sample to identify which diagnoses are new symptoms of PASC and not due to pre-existing comorbidities.

To determine what symptoms and diagnoses are occurring at higher rates after acute COVID-19 infection, the current study utilized four years of electronic hospital records (EHR) data both prior to and following acute SARS-CoV-2 infection. Using this analysis, the occurrence of diagnoses will be examined for each patient to identify whether the odds of a diagnosis after a positive PCR test for COVID-19 are greater than they were prior to the test.

## Methods

### Study design and participants

**Fig 1:**
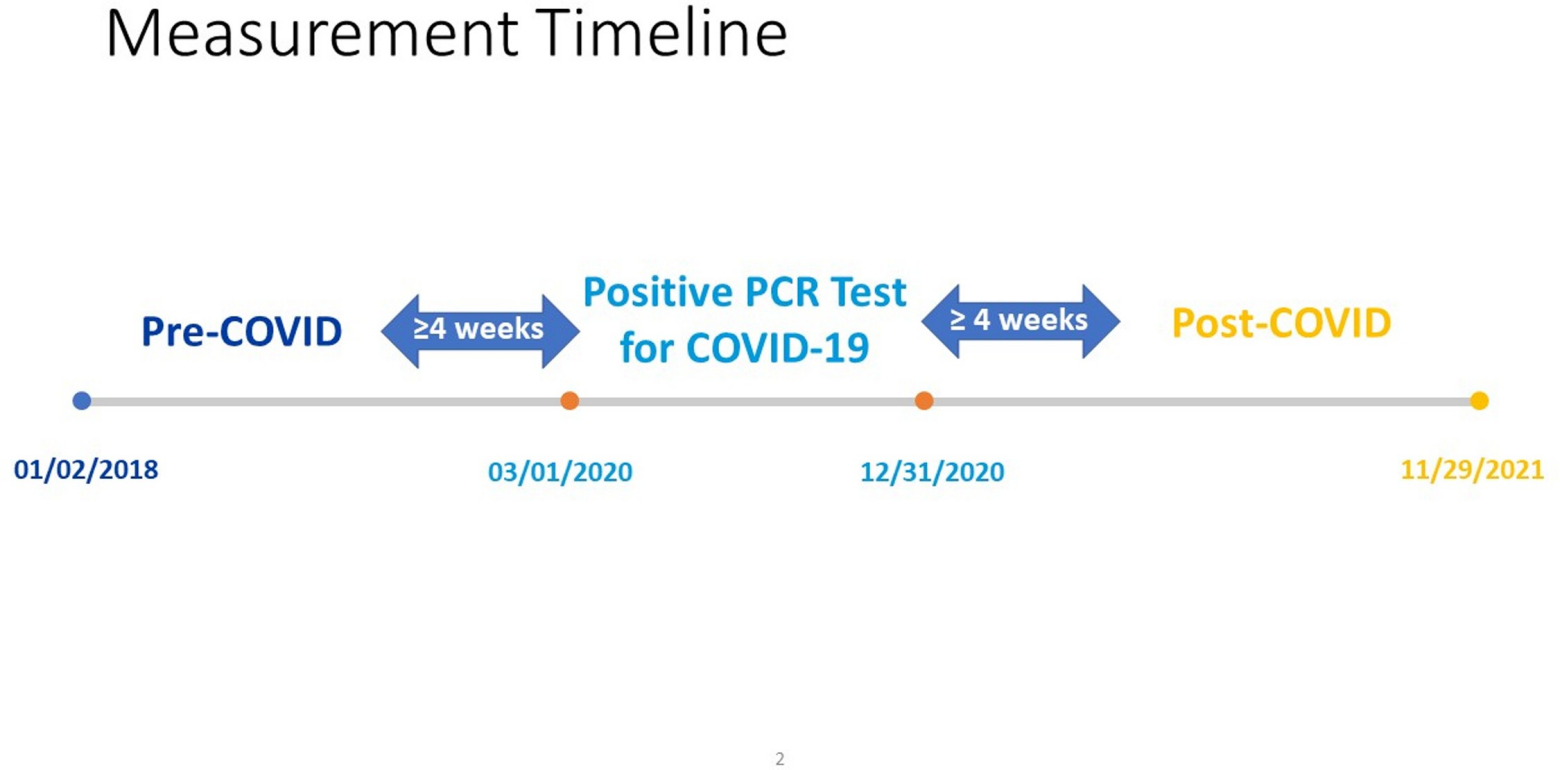
Measurement timeline for the study.

This retrospective study examined the frequencies of diagnostic categories before and after acute COVID-19 disease. The sample was comprised of patients who had a positive PCR test for SARS-CoV-2 between March 1, 2020 and December 31, 2020 and were treated at Northwell Health, a large academic healthcare system that comprises 23 hospitals/medical facilities serving New York City and the surrounding area. The New York city region was one of the epicenters of the COVID-19 pandemic in the United States [10] and a large volume of patients were tested and treated in the Northwell Health system. Follow-up for these patients was conducted through November 29, 2021. The earliest recorded encounter prior to the positive PCR test was January 2, 2018 (Fig 1). The Northwell Health institutional review board approved this observational analysis as minimal-risk research using data collected for routine clinical practice and waived the requirement for informed consent.

The Northwell Health electronic health record (EHR) system included records for 27,876 patients who had a positive PCR test for COVID-19 between March 1, 2020 and November 29, 2021 and had previous encounters in 2018 or 2019. Encounters were defined as either an inpatient or outpatient contact with a provider recorded in the Northwell Health EHR. Of this group, 4,901 (17.6%) patients had no other recorded encounters in the dataset besides their PCR and were excluded from the analysis. To allow for a follow-up period after positive PCR test and to reduce complications from the inclusion of vaccinated patients, we excluded an additional 9,886 (35.5%) patients whose first positive PCR test occurred on January 1^st^, 2021 or later. Another 56 (< .01%) patients were excluded for having encounters less than 4 weeks prior or following a positive PCR test. Patients with mortality due to initial infection due to COVID-19 were excluded from the analysis. Mortality during follow-up was not recorded, only the occurrence of diagnoses following PCR test. This yielded a final analysis sample of 13,033 patients who had encounters in the EHR both before and after a positive PCR test for COVID-19 (S1 Fig).

### Outcomes

To determine which categories of diagnoses could be the result of Post-Acute Sequelae of SARS-CoV-2 Infection (PASC) also known as “long COVID” [2,4], we reviewed the literature to identify potential consequences of COVID-19 illness. Prior research has suggested that inflammation from SARS-CoV-2 infection may lead to increased incidence of acute coronary syndrome (ACS), arrythmias, cardiomyopathy, coronary heart failure (CHF), myocarditis, pericarditis, stroke, and other cardiovascular complications among patients recovering from COVID-19 [11–14]. Damage to the lungs and abnormal coagulation resulting from acute SARS-CoV-2 infection has also been associated with subsequent bronchiectasis, cough, fatigue, pulmonary fibrosis, pulmonary embolism, and other pulmonary complications [15–19]. The damage to multiple organs seen in acute COVID-19 has also been linked with kidney injury [20,21], liver injury [22,23], pancreas injury [24,25], and injury to the spleen [26].

Neurological complications, including cognitive impairment, have also been reported among patients who have recovered from acute COVID-19 [27,28]. Other symptoms reported by patients following acute infection include fatigue, shortness of breath, loss of taste/smell, muscle/joint pain, cough, headache, dizziness, gastrointestinal distress, and trouble sleeping [28,29].

In addition to the physical consequences of COVID-19, research has also shown psychological/psychiatric of the disease as well. Anxiety and depression have been found to be significantly prevalent among patients following acute COVID-19 [30,31], though during periods of quarantine and isolation, levels of anxiety and depression were found in high prevalence among the general population [32]. COVID-19 infection has been linked to other potential psychiatric conditions as well [4,33].

Integrating this research, we identified 21 categories of diagnoses which could be attributed to PASC: ACS, Anxiety & Depression, Arrythmias, Bronchiectasis & Cough, CHF & Cardiomyopathy, Chest Pain, Cognitive Impairment, Dizziness & Headache, Dyspnea & Respiratory Failure, Fatigue, Kidney/Liver/Pancreas/Spleen Injury, Loss of Smell or Taste, Myositis & Musculoskeletal Pain/Stiffness, Nausea/Vomiting/Diarrhea, Other Psychiatric Disorder, Pericarditis & Myocarditis, Platelet/Clotting Dysfunctions, Pulmonary Embolism, Pulmonary Fibrosis, Sleep Disturbance, and Stroke.

**Fig 2:**
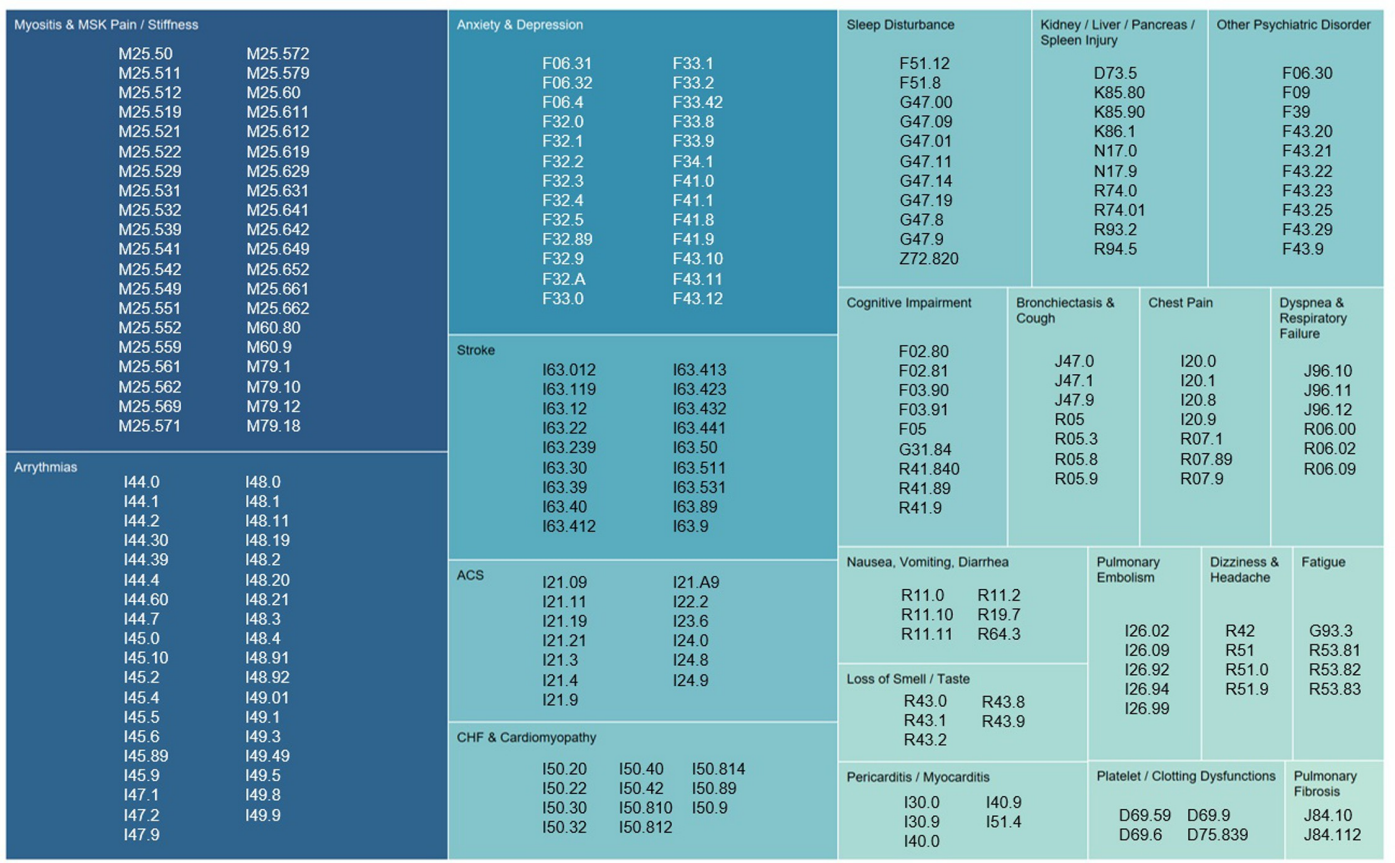
Outcome categories by ICD-10 code.

An illustration of how these diagnosis categories map on to their associated ICD-10 codes can be found in Fig 2.

### Statistical analysis

We summarized the frequencies (%) of diagnosis categories before and after positive PCR test for SARS-CoV-2, and employed conditional logistic regression models to estimate the odds of having a PASC related diagnosis along with the 95% confidence intervals (CIs). This allowed patients who had multiple diagnoses in the periods before and after positive PCR test to contribute to multiple regression outcomes. In using these conditional logistic regression model, we are able to compare the pre- and post-PCR diagnoses for each patient, thereby allowing each patient to be their own reference. As previous research has demonstrated that patient characteristics may influence outcomes of COVID-19 [34–36], exploratory stratified analyses were conducted to determine whether the odds of these diagnostic categories occurring following COVID-19 infection relative to visits occurring prior to infection varied by important demographics such as age, gender, ethnicity, and race.

## Results

**Fig 3:**
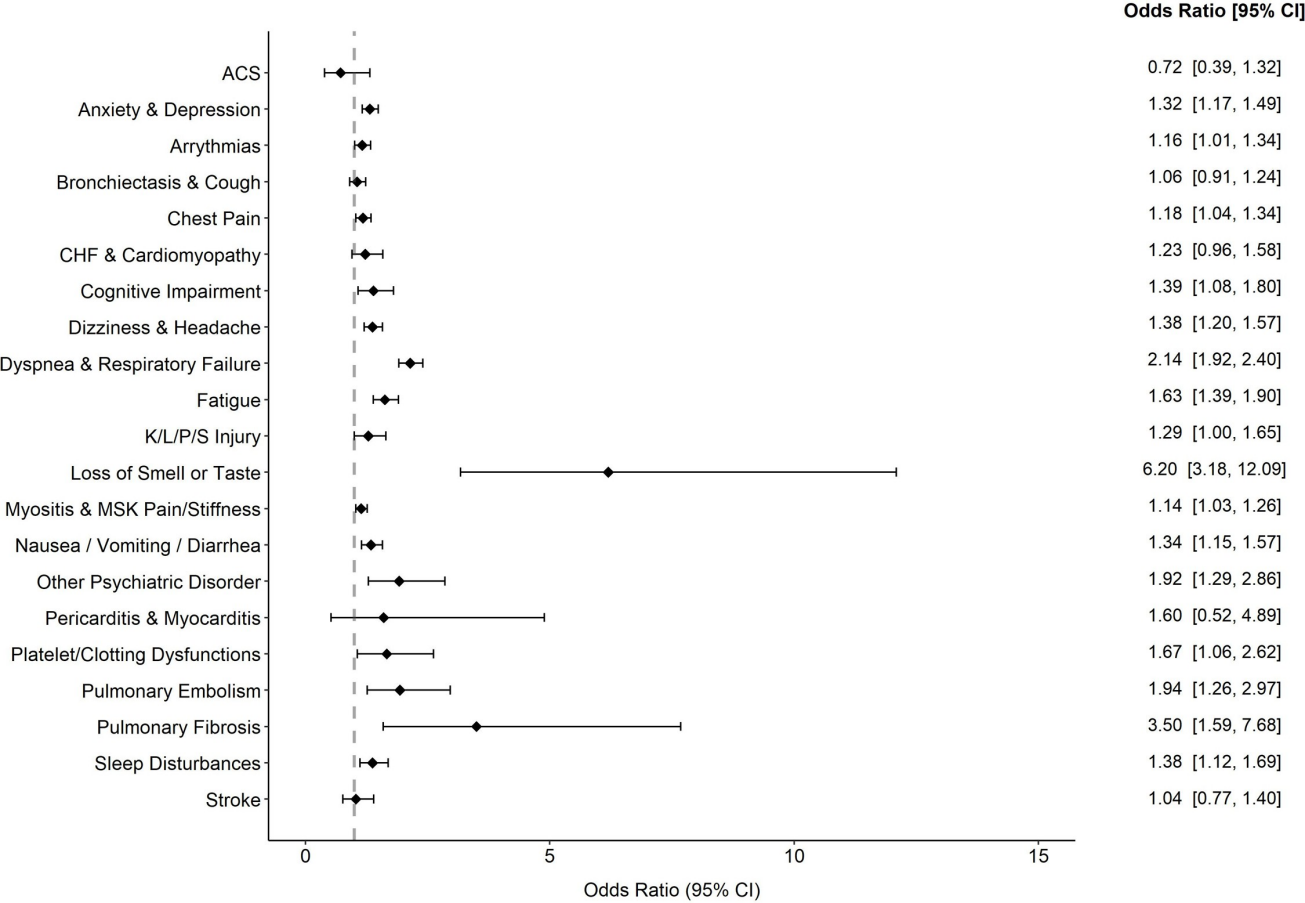
Odds ratios of symptoms and diagnoses following positive PCR test vs Pre PCR test. Note: Odds ratios above presented for conditional logistic regression analysis among 13,033 patients comparing symptoms and diagnoses recorded after positive PCR for COVID-19 relative to prior positive PCR.

Thirteen thousand and thirty-three patients had at least 1 inpatient/outpatient appointment before and after a positive polymerase chain reaction (PCR) test for COVID-19 between March 1, 2020, and December 31, 2020. Patient inpatient and outpatient encounters between January 2, 2018, and November 29, 2021, were examined to determine diagnoses before and after the PCR test. The median length of follow-up for patients was 33 weeks (approximately 8 months), with most of the sample being followed between 17 and 53 weeks after the PCR test. Sample characteristics and frequencies of symptoms and diagnoses before and after a positive PCR test are found in Table 1. After the positive PCR test, the most recorded diagnoses and symptoms were dyspnea and respiratory failure, myositis, musculoskeletal pain/stiffness, anxiety, and depression. Results from regression analyses showed increased odds of diagnosis for 15 of the 21 categories following positive PCR (Fig 3). Relative to pre-COVID, the diagnoses and symptoms with the greatest odds after a positive PCR test were loss of smell or taste, pulmonary fibrosis, and dyspnea/respiratory failure. Stratification of these analyses by age, gender, race, and ethnicity showed similar results (Supplemental Materials). Table 2 shows the median time diagnoses occurred following a positive PCR test. For all diagnoses, the median time was 33 weeks (interquartile range: 16 to 53 weeks). Diagnostic categories with the smallest median times between positive PCR test and subsequent diagnosis included pulmonary embolism (16 weeks), CHF and cardiomyopathy (25.5 weeks), and dyspnea and respiratory failure (27 weeks). Diagnostic categories with the longest median time following positive PCR test included cognitive impairment (43 weeks), bronchiectasis and cough (42 weeks), and other psychiatric disorder (40 weeks).

**Table 1:** Patients’ characteristics.

Patient Characteristics		Frequency N (%)	
***Full Sample***		***13*,*033 (100*.*0%)***	
Age	≤ 65	9,436 (72.0%)	
	> 65	3,597 (28.0%)	
Sex	Female	7,472 (57.0%)	
	Male	5,561 (43.0%)	
Race	Asian	606 (4.7%)	
	Black/African-American	1,909 (14.6%)	
	Other	2,740 (21.0%)	
	White	7,062 (54.2%)	
	Missing/Not Reported	716 (5.5%)	
Ethnicity	Hispanic/Latino	2,357 (18.1%)	
	Non-Hispanic	9,176 (70.4%)	
	Missing/Not Reported	1,500 (11.5%)	
**Diagnosis Categories**		**Pre-COVID**	**Post-COVID**
Acute Coronary Syndrome (ACS)		25 (0.19%)	18 (0.14%)
Anxiety & Depression		488 (3.74%)	629 (4.83%)
Arrythmias		413 (3.17%)	472 (3.62%)
Bronchiectasis & Cough		319 (2.45%)	337 (2.59%)
Chest Pain		437 (3.35%)	513 (3.94%)
Coronary Heart Failure (CHF) & Cardiomyopathy		115 (0.88)	140 (1.07%)
Cognitive Impairment		105 (0.81%)	144 (1.10%)
Dizziness & Headache		397 (3.05%)	533 (4.09%)
Dyspnea & Respiratory Failure		491 (3.77%)	997 (7.65%)
Fatigue		263 (2.02%)	422 (3.24%)
Kidney/Liver/Pancreas/Spleen Injury		116 (0.89%)	148 (1.14%)
Loss of Smell or Taste		10 (0.08%)	62 (0.48%)
Myositis & Musculoskeletal (MSK) Pain/Stiffness		822 (6.31%)	922 (7.07%)
Nausea/Vomiting/Diarrhea		279 (2.14%)	370 (2.84%)
Other Psychiatric Disorder		41 (0.31%)	75 (0.58%)
Pericarditis & Myocarditis		5 (0.04%)	8 (0.06%)
Platelet/Clotting Dysfunctions		32 (0.25%)	52 (0.40%)
Pulmonary Embolism		32 (0.25%)	62 (0.48%)
Pulmonary Fibrosis		8 (0.06%)	28 (0.21%)
Sleep Disturbances		156 (1.20%)	214 (1.64%)
Stroke		84 (0.64%)	87 (0.67%)

**Table 2:** Time to diagnosis following positive PCR test.

Overall Summary of Time Between Positive PCR Test and Diagnosis			
Minimum/Maximum	Median (IQR)		Mean (SD)
4 to 88	33 (16 to 53)		36.2 (22.8)
Symptom Group	Median (IQR)		Mean (SD)
ACS	34	(9.5, 40)	31.6 (22.4)
Anxiety and Depression	37	(19, 55)	38.9 (23.2)
Arrythmias	29.5	(14, 48.75)	33.4 (21.8)
Bronchiectasis and Cough	42	(19.5, 61.5)	41.5 (25.3)
Chest pain	30	(15, 53)	34.9 (22.9)
CHF and Cardiomyopathy	25.5	(15, 48)	32.1 (20.9)
Cognitive impairment	43	(23, 66)	44.2 (24.6)
Dizziness and Headache	32	(20, 54)	37.2 (22.1)
Dyspnea and Respiratory failure	27	(12, 46)	31.6 (21.9)
Fatigue	29	(16.5, 50)	34.8 (22.2)
Kidney/Liver/Pancreas/Spleen injury	32.5	(16, 53.25)	35.5 (22.2)
Loss of smell / Loss of Taste	27.5	(17, 38)	28.31 (16.95)
Myositis and MSK pain / stiffness	38	(20, 58)	40.10 (23.07)
Nausea, Vomiting, or Diarrhea	34	(17, 51)	36.08 (21.47)
Other psychiatric disorder	40	(20.25, 60.75)	40.81 (23.98)
Pericarditis / Myocarditis	36	(24, 47)	36.44 (24.52)
Platelet/clotting dysfunctions	32.5	(13, 56)	36.88 (24.85)
Pulmonary embolism	16	(10, 31.5)	23.39 (19.63)
Pulmonary fibrosis	32	(16, 40)	29.97 (16.91)
Sleep disturbance	32.5	(15, 49.25)	34.28 (21.67)
Stroke	32	(16.75, 52.25)	35.65 (21.76)

### Stratified analyses by age (≤65 and >65 years old)

As in the full cohort, loss of taste or smell, pulmonary fibrosis, dyspnea, and respiratory failure occurred more frequently after positive PCR than before (S2 Fig). However, cognitive impairment had greater odds of being diagnosed after acute COVID-19 infection among patients ≤65 years old [Odds Ratio (95% CI) = 2.80 (1.55 to 5.05)] than among patients >65 years old [OR (95% CI) = 1.14 (0.85 to 1.53)]. Arrythmias, pulmonary embolism, and chest pain also had greater odds of being diagnosed following positive PCR test among patients ≤65 years old than in patients >65 years old (S1 Table). In contrast, platelet and clotting dysfunctions were more frequently diagnosed after COVID-19 illness among patients >65 years old [OR (95% CI) = 2.86 (1.21 to 6.76)] than in patients ≤65 years old [OR (95% CI) = 1.30 (0.76 to 2.25)]. Other results were comparable between older and younger patients.

### Stratified analyses by gender (Male and Female)

Regressions examining diagnostic categories by patient gender showed similar patterns between the two groups. As in the full cohort, loss of smell or taste, pulmonary fibrosis, and dyspnea and respiratory failure were some of the most diagnosed categories after a positive PCR test for COVID-19 (S3 Fig). Male patients did show greater odds of having a pulmonary embolism diagnosis [OR (95% CI) = 2.33 (1.27 to 4.27)] compared to female patients [OR (95% CI) = 1.59 (0.87 to 2.91)]. The reverse was true for chest pain, cognitive impairment, myositis/MSK pain/stiffness, other psychiatric disorder, and sleep disturbances. For these diagnosis categories women showed higher odds following a positive PCR test than men did (S2 Table).

### Stratified analyses by ethnicity (Hispanic and Non-Hispanic)

In analyses examining odds of diagnostic categories by Hispanic ethnicity, the results were similar to the primary analysis with pulmonary fibrosis, and dyspnea and respiratory failure having higher odds of diagnosis following a positive PCR test (S3 Table and S4 Fig). Differences between groups included a higher odds of CHF/cardiomyopathy and cognitive impairment following PCR test among Hispanic patients than among non-Hispanic patients. Arrythmias, kidney/liver/pancreas/spleen injury, loss of smell or taste, other psychiatric disorder, and platelet/clotting dysfunction had greater odds of diagnosis following PCR test among non-Hispanic patients than Hispanic patients.

### Stratified analyses by race (African American, Asian, White, Other)

For analyses stratified by race, the sample sizes in each group impacted the precision of the estimates, i.e., generating wide confidence intervals. For example, the odds of cognitive impairment following a positive PCR test for COVID-19 among Asians was large [OR (95% CI) = 5.00 (0.58 to 42.80)]. However, some of the diagnoses with greater odds of diagnosis after positive PCR test across all racial groups were loss of taste or smell and dyspnea and respiratory failure (S4 Table and S5 Fig). Pulmonary fibrosis also had high odds of diagnosis following a positive PCR test for most groups, but no events were recorded in the Asian sub-group.

## Discussion

The 15 categories which demonstrated greater odds of diagnosis following SARS-CoV-2 infection match previous research identifying broad categories of PASC [1,2,9]. Included among these categories are pulmonary diseases (e.g. pulmonary fibrosis), cardiovascular conditions (arrythmia), patient-reported symptoms (e.g. cognitive impairment), and psychological conditions (e.g. anxiety & depression). The breadth of these diagnoses emphasizes the wide-reaching and long-lasting effects that COVID-19 has on patients. These findings reinforce that COVID-19 can exert long-lasting influence on multiple body systems. Further, our results showed that most patients were diagnosed with PASC symptoms between 16 and 53 weeks after their initial diagnosis. This supports the notion that these symptoms are not simply post-acute responses to COVID-19 occurring 3 to 12 weeks after infection [4].

The current research expands upon the literature by showing that these symptoms are more frequently detected following acute COVID-19 than before COVID-19, an acknowledged limitation of prior PASC research [5]. A recently published manuscript examined symptoms of PASC by comparing individuals with a positive PCR test to a matched cohort without a positive PCR [8]. Though this analysis matched the positive and negative cohorts based on participant characteristics and comorbid conditions (including kidney disease, chronic obstructive pulmonary disease, diabetes, HIV, pregnancy, and cancer), the analysis did not include pre-occurring symptoms associated with PASC. By accounting for pre-morbid diagnoses, the current study is able to better qualify that the symptoms and diagnoses detected are truly PASC rather than some alternative phenomenon. Further, our analyses provide a broad snapshot of the population as we were able to describe PASC among all patients who tested positive for COVID-19.

The current analyses also examine PASC stratified by important demographic characteristics. The goal of the stratified analyses was mainly exploratory, with no formal hypotheses or adjustment for multiple testing conducted. Due to this, the results should be interpreted cautiously and used to identify questions for future research, not to make conclusive statements about PASC based on differences in participant characteristics. Further, small sample sizes among different demographic and low numbers of diagnoses recorded may influence the interpretability of some findings. For example, the infrequent number of cases of pericarditis and myocarditis among Asian patients yielded large confidence intervals for our estimate of odds of PASC in this category. However, these findings suggest that the relative odds of particular diagnoses following COVID-19 differ by age, sex, ethnicity, and race. The underlying mechanisms for these differences will need to be examined by future research to determine how they may be addressed and treated.

An additional limitation is that the current analysis is a retrospective observational study utilizing data from the EHR. The use of EHR data in the analyses introduce several potential biases including differences in how individual physicians select and apply ICD-10 diagnoses. Each physician will apply their own judgement in identifying diagnoses and applying them in the EHR. Further, diagnoses may even be erroneously recorded due to physician error [37]. In addition, the current analysis combines EHR data from multiple disparate settings including inpatient care and primary practice. This may introduce potential biases in how diagnoses are applied in those settings. However, these limitations are common with studies using retrospective analyses of health records [38]. As this study is exploratory in nature, we believe the current results can be useful to guide future research despite potential sources of bias introduced by the use of EHR data.

## Conclusions

By accounting for diagnoses prior to SARS-CoV-2 infection, the findings of this manuscript support and strengthen prior research by showing that the diagnoses and symptoms attributed to PASC accurately reflect the long-term consequences of COVID-19 illness. However, this study is exploratory in nature. Additional research will be required to determine which diagnostic categories become chronic over more extended follow-up periods. As the severity of COVID-19 may influence PASC, additional research will be required to identify which diagnosis categories are more prevalent in mild versus severe illness. Further science is also required to identify the reasons and mechanisms for symptoms of PASC as well as explanations for demographic differences in the presentation of PASC. Without understanding how COVID-19 illness influences patient symptoms long after acute illness has faded, it will be extremely difficult to prevent, diagnose, and manage PASC.

## Supporting information

S1 Fig Exclusion criteria and sample size. (PNG)

S2 Fig Age-stratified odds of diagnostic category. (JPG)

S3 Fig Gender-stratified odds of diagnostic category. (JPG)

S4 Fig Ethnicity-stratified odds of diagnostic category. (JPG)

S5 Fig Race-stratified odds of diagnostic category. (JPG)

S1 Table Age-stratified odds of diagnostic category. (PDF)

S2 Table Gender-stratified odds of diagnostic category. (PDF)

S3 Table Ethnicity-stratified odds of diagnostic category. (PDF)

S4 Table Race-stratified odds of diagnostic category. (PDF)

## References

[1] Nasserie T, Hittle M, Goodman SN. Assessment of the frequency and variety of persistent symptoms among patients with COVID-19: a systematic review. JAMA network open 2021;4(5):e2111417–e17. doi: 10.1001/jamanetworkopen.2021.11417 34037731 PMC8155823

[2] Al-Aly Z, Xie Y, Bowe B. High-dimensional characterization of post-acute sequelae of COVID-19. Nature 2021;594(7862):259–64. doi: 10.1038/s41586-021-03553-9 33887749

[3] Crook H, Raza S, Nowell J, et al. Long covid—mechanisms, risk factors, and management. bmj 2021;374. doi: 10.1136/bmj.n1648 34312178

[4] Raveendran A, Jayadevan R, Sashidharan S. Long COVID: an overview. Diabetes & Metabolic Syndrome: Clinical Research & Reviews 2021;15(3):869–75. doi: 10.1016/j.dsx.2021.04.007 33892403 PMC8056514

[5] Munblit D, Nicholson TR, Needham DM, et al. Studying the post-COVID-19 condition: research challenges, strategies, and importance of Core Outcome Set development. BMC medicine 2022;20(1):1–13. 35114994 10.1186/s12916-021-02222-y PMC8813480

[6] Estiri H, Strasser ZH, Brat GA, et al. Evolving phenotypes of non-hospitalized patients that indicate long COVID. BMC medicine 2021;19(1):1–10. 34565368 10.1186/s12916-021-02115-0 PMC8474909

[7] Nandasena H, Pathirathna M, Atapattu A, et al. Quality of life of COVID 19 patients after discharge: Systematic review. PloS one 2022;17(2):e0263941. doi: 10.1371/journal.pone.0263941 35171956 PMC8849513

[8] Horberg MA, Watson E, Bhatia M, et al. Post-acute sequelae of SARS-CoV-2 with clinical condition definitions and comparison in a matched cohort. Nature communications 2022;13(1):5822. doi: 10.1038/s41467-022-33573-6 36224218 PMC9556630

[9] van Kessel SA, Olde Hartman TC, Lucassen PL, et al. Post-acute and long-COVID-19 symptoms in patients with mild diseases: a systematic review. Family practice 2022;39(1):159–67. doi: 10.1093/fampra/cmab076 34268556 PMC8414057

[10] Goyal P, Choi JJ, Pinheiro LC, et al. Clinical Characteristics of Covid-19 in New York City. N Engl J Med 2020;382(24):2372–74. doi: 10.1056/NEJMc2010419 [published Online First: 2020/04/18]. 32302078 PMC7182018

[11] Grzegorowska O, Lorkowski J. Possible correlations between atherosclerosis, acute coronary syndromes and COVID-19. Journal of Clinical Medicine 2020;9(11):3746. doi: 10.3390/jcm9113746 33233333 PMC7700642

[12] Lee CC, Ali K, Connell D, et al. COVID-19-associated cardiovascular complications. Diseases 2021;9(3):47. doi: 10.3390/diseases9030047 34209705 PMC8293160

[13] Patone M, Mei XW, Handunnetthi L, et al. Risks of myocarditis, pericarditis, and cardiac arrhythmias associated with COVID-19 vaccination or SARS-CoV-2 infection. Nature medicine 2022;28(2):410–22. doi: 10.1038/s41591-021-01630-0 34907393 PMC8863574

[14] Long B, Brady WJ, Koyfman A, et al. Cardiovascular complications in COVID-19. The American journal of emergency medicine 2020;38(7):1504–07. doi: 10.1016/j.ajem.2020.04.048 32317203 PMC7165109

[15] Martinez-Garcia MA, Aksamit TR, Aliberti S. Bronchiectasis as a Long-Term Consequence of SARS-COVID-19 Pneumonia: Future Studies are Needed. Archivos de bronconeumologia 2021;57(12):739–40. 10.1016/j.arbr.2021.04.017 PMC8634802 34866750

[16] Daher A, Balfanz P, Cornelissen C, et al. Follow up of patients with severe coronavirus disease 2019 (COVID-19): Pulmonary and extrapulmonary disease sequelae. Respiratory medicine 2020;174:106197. doi: 10.1016/j.rmed.2020.106197 33120193 PMC7573668

[17] Sakr Y, Giovini M, Leone M, et al. Pulmonary embolism in patients with coronavirus disease-2019 (COVID-19) pneumonia: a narrative review. Annals of intensive care 2020;10(1):1–13. 32953201 10.1186/s13613-020-00741-0 PMC7492788

[18] Akel T, Qaqa F, Abuarqoub A, et al. Pulmonary embolism: a complication of COVID 19 infection. Thrombosis research 2020;193:79–82. doi: 10.1016/j.thromres.2020.05.033 32526545 PMC7247481

[19] Spagnolo P, Balestro E, Aliberti S, et al. Pulmonary fibrosis secondary to COVID-19: a call to arms? The Lancet Respiratory Medicine 2020;8(8):750–52. doi: 10.1016/S2213-2600(20)30222-8 32422177 PMC7228737

[20] Ahmadian E, Hosseiniyan Khatibi SM, Razi Soofiyani S, et al. Covid‐19 and kidney injury: pathophysiology and molecular mechanisms. Reviews in medical virology 2021;31(3):e2176. doi: 10.1002/rmv.2176 33022818 PMC7646060

[21] Ronco C, Reis T, Husain-Syed F. Management of acute kidney injury in patients with COVID-19. The Lancet Respiratory Medicine 2020;8(7):738–42. doi: 10.1016/S2213-2600(20)30229-0 32416769 PMC7255232

[22] Alqahtani SA, Schattenberg JM. Liver injury in COVID-19: The current evidence. United European gastroenterology journal 2020;8(5):509–19. doi: 10.1177/2050640620924157 32450787 PMC7268949

[23] Nardo AD, Schneeweiss‐Gleixner M, Bakail M, et al. Pathophysiological mechanisms of liver injury in COVID‐19. Liver International 2021;41(1):20–32. doi: 10.1111/liv.14730 33190346 PMC7753756

[24] Wang F, Wang H, Fan J, et al. Pancreatic injury patterns in patients with coronavirus disease 19 pneumonia. Gastroenterology 2020;159(1):367–70. doi: 10.1053/j.gastro.2020.03.055 32247022 PMC7118654

[25] Mukherjee R, Smith A, Sutton R. Covid-19-related pancreatic injury. Journal of British Surgery 2020;107(7):e190–e90. doi: 10.1002/bjs.11645 32352160 PMC7267547

[26] Feng Z, Diao B, Wang R, et al. The novel severe acute respiratory syndrome coronavirus 2 (SARS-CoV-2) directly decimates human spleens and lymph nodes. MedRxiv 2020.

[27] Bridwell R, Long B, Gottlieb M. Neurologic complications of COVID-19. The American journal of emergency medicine 2020;38(7):1549. e3-49. e7. doi: 10.1016/j.ajem.2020.05.024 32425321 PMC7229718

[28] Hirschtick JL, Titus AR, Slocum E, et al. Population-based estimates of post-acute sequelae of SARS-CoV-2 infection (PASC) prevalence and characteristics. Clinical Infectious Diseases 2021. 10.1093/cid/ciab408 PMC8240848 34007978

[29] Wang L, Foer D, MacPhaul E, et al. PASCLex: A comprehensive post-acute sequelae of COVID-19 (PASC) symptom lexicon derived from electronic health record clinical notes. Journal of biomedical informatics 2022;125:103951. doi: 10.1016/j.jbi.2021.103951 34785382 PMC8590503

[30] Tomasoni D, Bai F, Castoldi R, et al. Anxiety and depression symptoms after virological clearance of COVID‐19: a cross‐sectional study in Milan, Italy. Journal of medical virology 2021;93(2):1175–79. doi: 10.1002/jmv.26459 32841387 PMC7461061

[31] Renaud-Charest O, Lui LM, Eskander S, et al. Onset and frequency of depression in post-COVID-19 syndrome: A systematic review. Journal of Psychiatric Research 2021;144:129–37. doi: 10.1016/j.jpsychires.2021.09.054 34619491 PMC8482840

[32] Tang F, Liang J, Zhang H, et al. COVID-19 related depression and anxiety among quarantined respondents. Psychology & health 2021;36(2):164–78. doi: 10.1080/08870446.2020.1782410 32567952

[33] Fiorillo A, Gorwood P. The consequences of the COVID-19 pandemic on mental health and implications for clinical practice. European Psychiatry 2020;63(1). doi: 10.1192/j.eurpsy.2020.35 32234102 PMC7156565

[34] Ogedegbe G, Ravenell J, Adhikari S, et al. Assessment of racial/ethnic disparities in hospitalization and mortality in patients with COVID-19 in New York City. JAMA network open 2020;3(12):e2026881–e81. doi: 10.1001/jamanetworkopen.2020.26881 33275153 PMC7718605

[35] Levy TJ, Richardson S, Coppa K, et al. Development and validation of a survival calculator for hospitalized patients with COVID-19. MedRxiv 2020. doi: 10.1101/2020.04.22.20075416 32511640 PMC7276996

[36] Mountantonakis SE, Epstein LM, Coleman K, et al. The association of structural inequities and race with out-of-hospital sudden death during the COVID-19 pandemic. Circulation: Arrhythmia and Electrophysiology 2021;14(5):e009646. doi: 10.1161/CIRCEP.120.009646 33835821 PMC8136460

[37] Singh H, Giardina TD, Meyer AN, et al. Types and origins of diagnostic errors in primary care settings. JAMA internal medicine 2013;173(6):418–25. doi: 10.1001/jamainternmed.2013.2777 23440149 PMC3690001

[38] Worster A, Haines T. Advanced statistics: understanding medical record review (MRR) studies. Academic emergency medicine 2004;11(2):187–92. 14759964

