# Supplementary figures and images for: Before and after COVID-19: Changes in symptoms and diagnoses in 13,033 adults
Source: PLoS One. 2024 Mar 8;19(3):e0286371. doi: 10.1371/journal.pone.0286371 (PMC10923490; doi:10.1371/journal.pone.0286371)

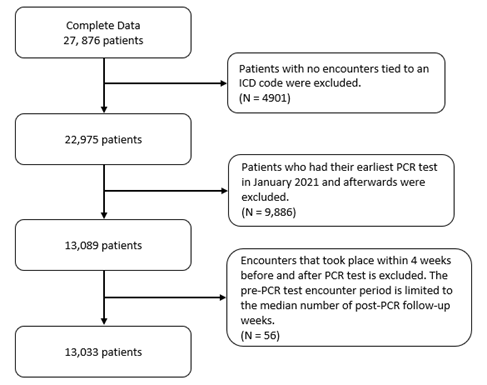

Supplement: S1 Fig — (PNG) [file pone.0286371.s001.png]

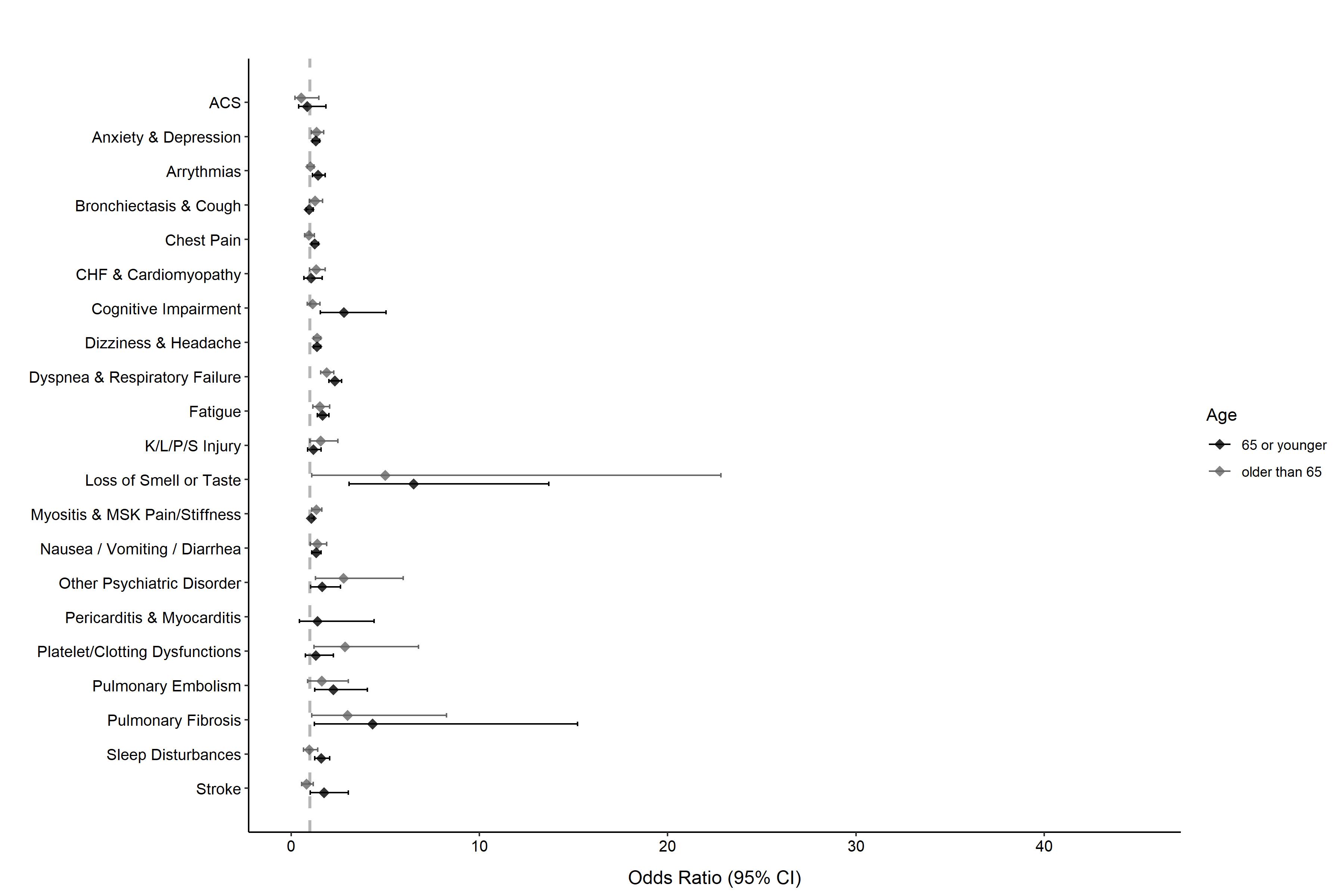

Supplement: S2 Fig — (JPG) [file pone.0286371.s002.jpg]

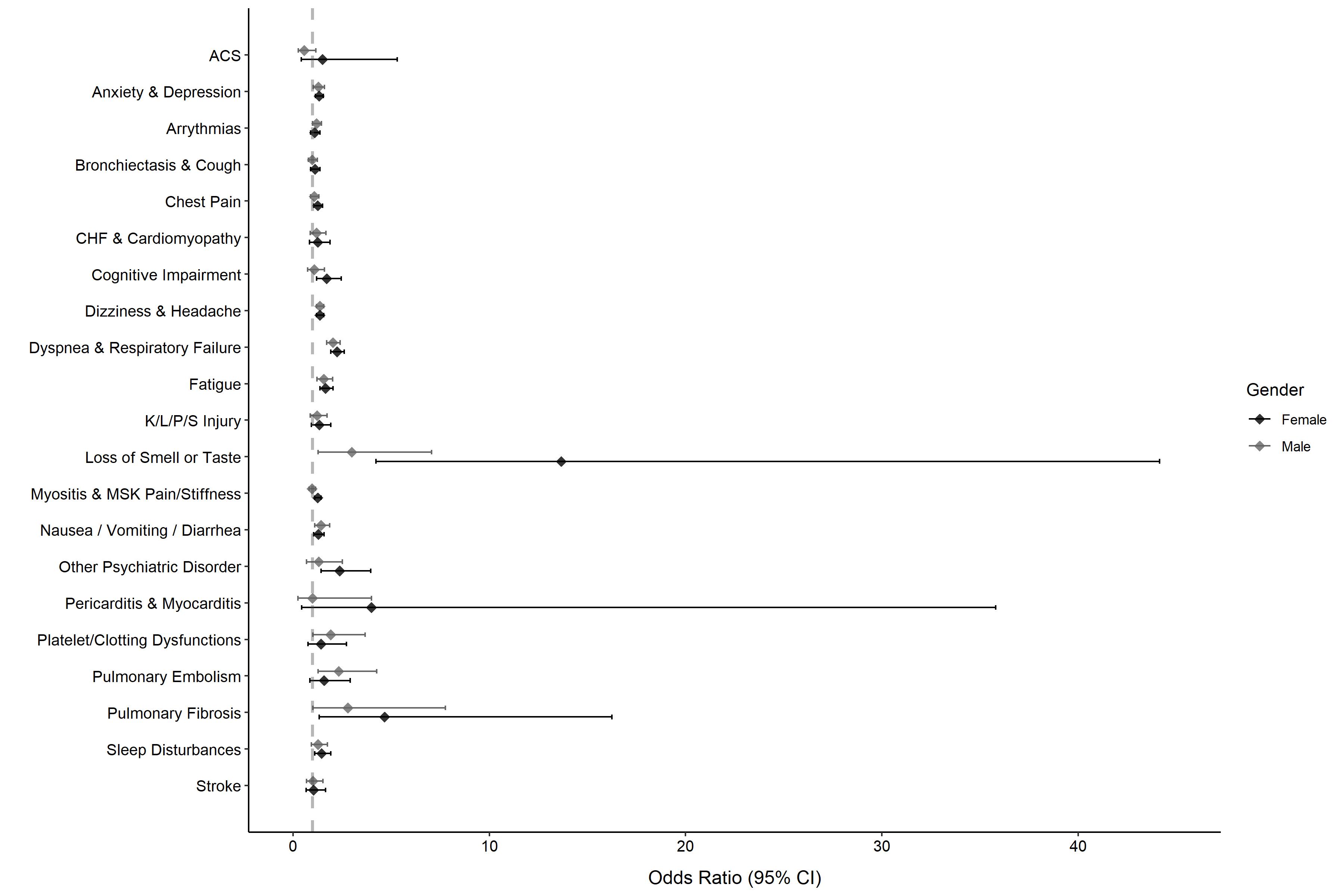

Supplement: S3 Fig — (JPG) [file pone.0286371.s003.jpg]

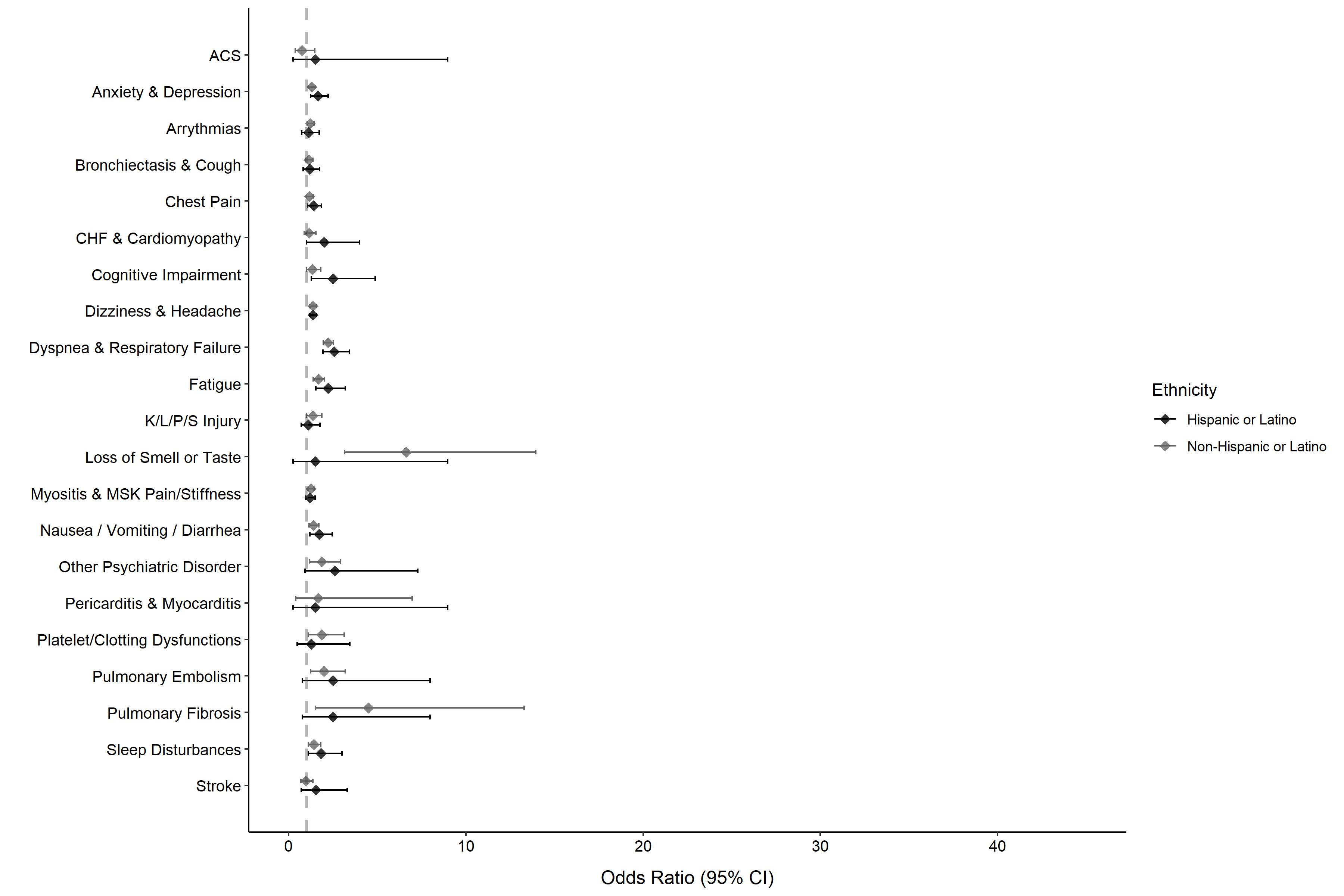

Supplement: S4 Fig — (JPG) [file pone.0286371.s004.jpg]

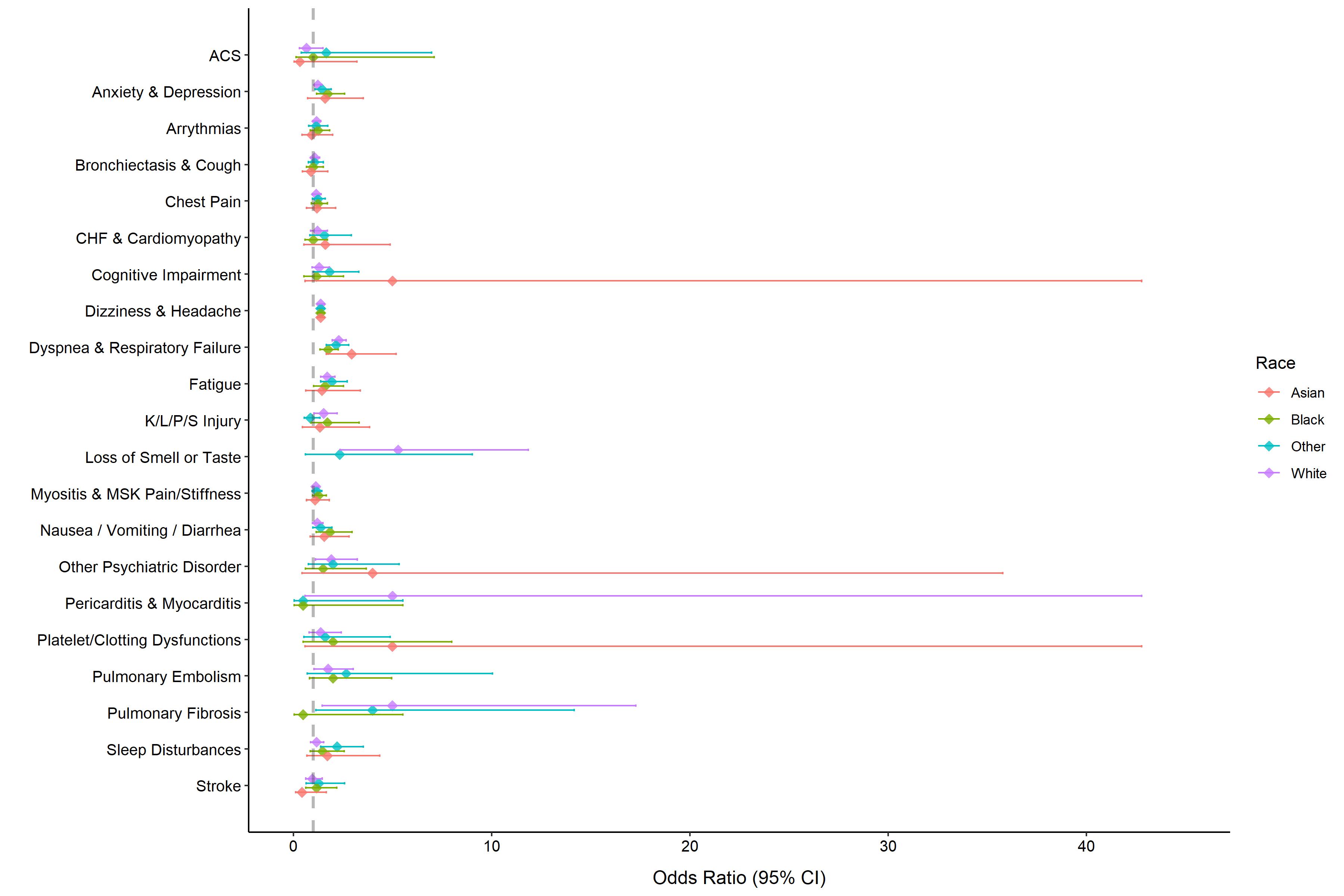

Supplement: S5 Fig — (JPG) [file pone.0286371.s005.jpg]
