# Supplementary material for: Before and after COVID-19: Changes in symptoms and diagnoses in 13,033 adults
Source: PLoS One. 2024 Mar 8;19(3):e0286371. doi: 10.1371/journal.pone.0286371 (PMC10923490; doi:10.1371/journal.pone.0286371)
Supplement: S3 Table — (PDF) [file pone.0286371.s008.pdf]

Supplemental Table 3. Ethnicity-stratified Odds of Diagnostic Category

	**Hispanic or Latinx**	**Not Hispanic or Latinx**
**Diagnoses**	**Odds Ratio (95% CI)**	**Odds Ratio (95% CI)**
Acute Coronary Syndrome (ACS)	1.50 (0.25, 8.98)	0.75 (0.38, 1.47)
Anxiety & Depression	1.66 (1.24, 2.23)	1.31 (1.13, 1.51)
Arrythmias	1.13 (0.74, 1.72)	1.22 (1.05, 1.43)
Bronchiectasis & Cough	1.20 (0.82, 1.75)	1.14 (0.95, 1.37)
Chest Pain	1.41 (1.08, 1.85)	1.18 (1.01, 1.38)
CHF & Cardiomyopathy	2.00 (1.00, 4.00)	1.16 (0.88, 1.54)
Cognitive Impairment	2.50 (1.28, 4.88)	1.35 (1.00, 1.82)
Dizziness & Headache	1.73 (1.31, 2.29)	1.36 (1.15, 1.61)
Dyspnea & Respiratory Failure	2.58 (1.94, 3.42)	2.22 (1.95, 2.53)
Fatigue	2.22 (1.54, 3.21)	1.68 (1.39, 2.03)
Kidney/Liver/Pancreas/Spleen Injury	1.12 (0.70, 1.78)	1.38 (1.02, 1.86)
Loss of Smell or Taste	1.50 (0.25, 8.98)	6.63 (3.15, 13.93)
Myositis & Musculoskeletal Pain/Stiffness	1.20 (0.97, 1.49)	1.26 (1.11, 1.42)
Nausea/Vomiting/Diarrhea	1.72 (1.20, 2.47)	1.40 (1.16, 1.70)
Other Psychiatric Disorder	2.60 (0.93, 7.29)	1.86 (1.19, 2.92)
Pericarditis & Myocarditis	1.50 (0.25, 8.98)	1.67 (0.40, 6.97)
Platelet/Clotting Dysfunctions	1.29 (0.48, 3.45)	1.86 (1.11, 3.13)
Pulmonary Embolism	2.50 (0.78, 7.97)	2.00 (1.25, 3.20)
Pulmonary Fibrosis	2.50 (0.78, 7.97)	4.50 (1.52, 13.30)
Sleep Disturbances	1.83 (1.12, 3.02)	1.42 (1.11, 1.82)
Stroke	1.55 (0.72, 3.30)	0.97 (0.69, 1.37)
