# Supplementary material for: Before and after COVID-19: Changes in symptoms and diagnoses in 13,033 adults
Source: PLoS One. 2024 Mar 8;19(3):e0286371. doi: 10.1371/journal.pone.0286371 (PMC10923490; doi:10.1371/journal.pone.0286371)
Supplement: S4 Table — (PDF) [file pone.0286371.s009.pdf]

***Supplemental Table 4.*** Race-stratified Odds of Diagnostic Category

| | African American / Black | Asian | White | Other |
|---|---|---|---|---|
| **Diagnosis Categories** | **Odds Ratio (95% CI)** | **Odds Ratio (95% CI)** | **Odds Ratio (95% CI)** | **Odds Ratio (95% CI)** |
| Acute Coronary Syndrome (ACS) | 1.00 (0.14, 7.10) | 0.33 (0.04, 3.21) | 0.67 (0.30, 1.48) | 1.67 (0.40, 6.97) |
| Anxiety & Depression | 1.74 (1.17, 2.59) | 1.60 (0.73, 3.53) | 1.24 (1.06, 1.44) | 1.45 (1.09, 1.91) |
| Arrythmias | 1.25 (0.86, 1.83) | 0.93 (0.44, 1.98) | 1.17 (0.98, 1.38) | 1.16 (0.77, 1.74) |
| Bronchiectasis & Cough | 1.00 (0.66, 1.52) | 0.89 (0.45, 1.74) | 1.08 (0.87, 1.33) | 1.07 (0.75, 1.52) |
| Chest Pain | 1.25 (0.91, 1.72) | 1.19 (0.67, 2.13) | 1.16 (0.96, 1.39) | 1.24 (0.96, 1.61) |
| CHF & Cardiomyopathy | 1.00 (0.58, 1.72) | 1.60 (0.52, 4.89) | 1.22 (0.87, 1.71) | 1.56 (0.83, 2.93) |
| Cognitive Impairment | 1.17 (0.54, 2.52) | 5.00 (0.58, 42.80) | 1.30 (0.94, 1.81) | 1.82 (1.01, 3.30) |
| Dizziness & Headache | 1.20 (0.84, 1.73) | 1.14 (0.64, 2.02) | 1.36 (1.12, 1.64) | 1.49 (1.14, 1.95) |
| Dyspnea & Respiratory Failure | 1.75 (1.34, 2.27) | 2.94 (1.67, 5.18) | 2.29 (1.96, 2.67) | 2.15 (1.67, 2.79) |
| Fatigue | 1.60 (1.01, 2.53) | 1.44 (0.62, 3.38) | 1.71 (1.39, 2.10) | 1.94 (1.39, 2.72) |
| Kidney/Liver/Pancreas/Spleen Injury | 1.71 (0.89, 3.31) | 1.33 (0.46, 3.84) | 1.52 (1.05, 2.21) | 0.86 (0.55, 1.34) |
| Loss of Smell or Taste | 1.62e+09 (0, Inf) | 1.62e+09 (0, Inf) | 5.29 (2.36, 11.86) | 2.33 (0.60, 9.02) |
| Myositis & Musculoskeletal Pain/Stiffness | 1.27 (0.97, 1.66) | 1.10 (0.67, 1.80) | 1.13 (0.98, 1.29) | 1.18 (0.96, 1.45) |
| Nausea/Vomiting/Diarrhea | 1.85 (1.16, 2.96) | 1.56 (0.86, 2.81) | 1.20 (0.97, 1.49) | 1.38 (0.98, 1.94) |
| Other Psychiatric Disorder | 1.50 (0.61, 3.67) | 4.00 (0.45, 35.79) | 1.91 (1.12, 3.23) | 2.00 (0.75, 5.33) |
| Pericarditis & Myocarditis | 0.50 (0.05, 5.51) | 1.62e+09 (0, Inf) | 5.00 (0.58, 42.80) | 0.50 (0.05, 5.51) |
| Platelet/Clotting Dysfunctions | 2.00 (0.50, 8.00) | 5.00 (0.58, 42.80) | 1.38 (0.79, 2.42) | 1.60 (0.52, 4.89) |
| Pulmonary Embolism | 2.00 (0.81, 4.96) | 1.62e+09 (0, Inf) | 1.76 (1.03, 3.01) | 2.67 (0.71, 10.05) |
| Pulmonary Fibrosis | 0.50 (0.05, 5.51) | - | 5.00 (1.45, 17.27) | 4.00 (1.13, 14.18) |
| Sleep Disturbances | 1.48 (0.85, 2.57) | 1.71 (0.68, 4.35) | 1.16 (0.88, 1.54) | 2.20 (1.37, 3.53) |
| Stroke | 1.17 (0.62, 2.19) | 0.43 (0.11, 1.66) | 0.95 (0.62, 1.46) | 1.29 (0.64, 2.59) |
